# Lifetime Prevalence of Abortion and Risk Factors in Women: Evidence from a Cohort Study

**DOI:** 10.1155/2020/4871494

**Published:** 2020-04-27

**Authors:** Mehdi Moradinazar, Farid Najafi, Zeinab Moradi Nazar, Behrooz Hamzeh, Yahya Pasdar, Ebrahim Shakiba

**Affiliations:** Research Center for Environmental Determinants of Health (RCEDH), Health Institute, Kermanshah University of Medical Sciences, Kermanshah, Iran

## Abstract

**Background:**

10-20% of pregnancies end due to spontaneous abortions. In recent years, nondocumentary evidence has been indicative of an increase in the prevalence of nonspontaneous abortions in Iran, especially in the Kurdish regions. The aim of this study is to assess the lifetime prevalence of spontaneous abortions and factors affecting spontaneous abortion in women 35-65 years old.

**Method:**

Data from the recruitment phase of Ravansar Non-Communicable Disease (RaNCD) cohort study was used. All of the 4831 married women 35-65 years old and with history of pregnancy were included in this study. In order to determine the abortion ratio, the number of abortions was divided by the number of live births, and multiple logistic regression analysis was applied to determine associated factors affecting abortion.

**Results:**

About 25.7% of women had a history of spontaneous abortion. The abortion ratio in women was 0.10. The abortion ratio in women with secondary education, first pregnancy and marriage age at ≥26, socioeconomic condition, and hyperthyroid and diabetes was high while the abortion ratio of women with high physical activity and BMI < 18.9 or residents of rural area was low. After assessing the effective variables, it was found that women with high blood pressure have 63% less odds for nonspontaneous abortion, which is statistically significant (*p* value < 0.05).

**Conclusion:**

Considering the effect of factors such as level of education, older age at the first marriage, and age at the first pregnancy on increased chance of spontaneous abortion, measures should be taken to take more care for these people.

## 1. Introduction

Spontaneous abortion is one of the most common complications of pregnancy [1]. In general, expulsion of an embryo or fetus before it reaches a stable stage of life is called abortion [2]. Studies indicate that the incidence of spontaneous abortion is between 10 and 20% [3–5]. It should also be noted that most of the spontaneous abortions occur in the early weeks of pregnancy, and therefore, it can be confused with menstrual bleeding [1, 6, 7]. Generally, it is very difficult to determine the rate of spontaneous and unwanted abortions because in countries where legal abortion is prohibited, there is a possibility of false report. Besides, the study of spontaneous abortion in low- and middle-income countries is also very challenging because most abortions have not been reported to and recorded in their official health system [8].

In more than half of the cases, the causes of abortion have been genetic disorders and chromosomal abnormalities [9, 10]. Nevertheless, other factors affecting abortion are as follows: uterine abnormalities [11, 12], infectious diseases and untreated diseases of the mother [1, 4], the age of the mother during pregnancy, previous history of abortion [1, 13, 14], age at the first menstruation [15], menstrual disorders [1], use of contraceptive drugs [1, 14], BMI > 25 kg/m^2^ [16–19], environmental conditions and mother's lifestyle such as smoking [20, 21] and use of caffeine [12, 13], being exposed to cigarette smoke [22, 23], stress [12, 24], exposure to mobile phone radiation [25], and low socioeconomic and employment status [26], which are effective in the occurrence of abortion.

Abortion is a distressing experience that affects the mother in a variety of ways by influencing on emotional status that can finally result in psychological disorders such as depression [27]. Although the prevalence of maternal abortion and miscarriage in Iran is stable, the number of new cases is slightly increasing. In fact, Iran is now facing with a decrease in population growth plus which is partly attributed to the increase in number of divorce and decrease in number of marriage among young men and women. While the nation is now trying to stabilize the growth of the population, a clear feature about the burden of abortion can help to understand the whole scenario. On the other hand, abortion is closely related to the cultural and religious factors, and Iran is a multicultural country with different ethnicities. Kurdish people mostly inhabited in western part of Iran with an integrated culture and life style. This study seeks to determine the lifetime prevalence of abortion and its risk factors in women 35-65 years old who have participated in the first cohort study among Kurdish people named Ravansar Non-Communicable Disease (RaNCD) cohort study.

## 2. Methodology

### 2.1. The Study Population

This cross-sectional study was conducted based on the population recruited for the RaNCD cohort study—a member of centers participated in Prospective Epidemiological Research Studies in Iran (PERSIAN). The recruitment phase began in November 2014 and ended in February 2017. During the course of this research, 10065 subjects willingly participated and signed the written informed consent letter. Further details have been presented elsewhere [28, 29].

### 2.2. Inclusion and Exclusion Criteria

Among all participating women, those with history of pregnancy were selected. In the RaNCD cohort study, the inclusion criteria for women were willingness to participate and complete the research, providing the signed written informed consent letter, and being capable of communication with the research team. For the purpose of this study, we excluded those who had no history of marriage and pregnancy.

### 2.3. Definition and Measurements

Socioeconomic status (SES), the main variable indicative of economic status of the family, was calculated by principal component analysis (PCA) and considered the subjects' wealth and social characteristics. Accordingly, the studied population was categorized into 5 quintiles: the poorest, the poor, the middle class, the rich, and the richest [30]. BIA device (InBody 770 BIOSPACE, Korea) was used for weight measurement. The heights measured with 0.1 accuracy using stadiometer [31]. A 19-item inventory related to light, moderate, and severe physical activities was used to collect information about the subjects' physical activity, and then, the Metabolic Equivalent of Task (MET) rate of each activity was obtained based on Compendium of Physical Activities to calculate daily MET rates of each participant. Physical activity levels were classified as low (MET 24-36.5 hours per week), moderate (MET 36.6-44.9 hours per week), and heavy (MET ≥45 hours per week) [32]. To measure the quality of nutrition, Healthy Eating Index (HEI)—based on the guidelines in 2015—was categorized into five groups [33]. In this study, any self-reported pregnancy ended spontaneously before week 20 is regarded as abortion. In order to determine the abortion ratio, the number of abortion was divided by the number of live births.

### 2.4. Statistical Methods

Continuous variables were mentioned as mean ± standard deviation, and qualitative variables were measured by frequency (%). In order to investigate about the risk factors of abortion, at first, a univariate logistic regression analysis was performed. Then, variables with *p* < 0.3 were entered in the multiple model. Thereafter, variables with *p* < 0.05 were kept, and other variables were excluded using a stepwise (Backward) method. In all of the analyses, missing values were deleted (less than 1%). All the analyses were performed using the STATA V.14 (STATA Corp LLC) software. *P* values < 0.05 were considered as statistically significant.

## 3. Results

From of 4831 women participants, 2083 (58%) of them were urban residents, and the rest were rural residents. 3472 (72.2%) of them experienced their first menstrual bleeding when they were between 13 and 16 years old. The education level of 2202 (45.9%) of the participants was between 1 and 5 years, and 3608 (74.8%) women had no consanguineous marriage. In total, 1241 (25.7%) had at least one spontaneous abortion during her life.

**Figure 1:**
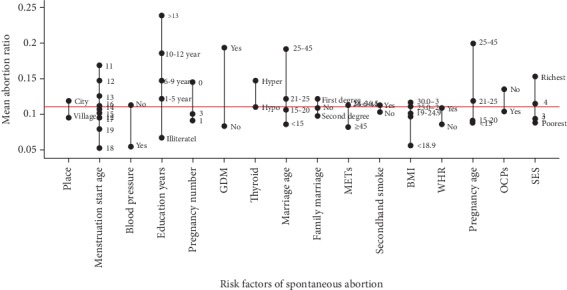
Mean abortion ratio based on the studied variables in the participants of RaNCD.

After adjustment for other variables, with the increase in the number of pregnancies, the risk for spontaneous abortion also increased; i.e., the odds for abortion in studied women who experienced more than 6 pregnancies was 8.3 (6.6-10.5) times significantly more than those with 1-3 pregnancies. The odds of abortion in women who married after 26 years old was 1.6 (1.02-2.4) times significantly more than the other. In addition, the risk of abortion in women who had their first pregnancy at age greater than 26 years old was 1.9 (1.3-2. 8) times significantly more than the others. Education level was an effective factor in spontaneous fetus abortion; i.e., with an increase in education level, the risk of abortion increased (Table 1). The highest abortion ratio was witnessed in women with secondary education (Figure 1).

From the total of women with past history of abortion, 24.8% had been using contraceptive pills. After adjusting the variables, it was shown that the use of pills is a protective factor for spontaneous abortion. Therefore, women who had been taking contraceptives pills had 22% less risk of abortion compared to those who had been using other contraceptive method which was statistically significant. Secondhand smoking nonsignificantly increased the odds ratio of abortion = 1.1 (95% C.I: 1.0-1.3). People who had the highest SES were in the greater risk of abortion (Table 1).

Compared to others, women with secondary education and those with marriage and first pregnancy age greater than 26 years, higher SES, past history of hyperthyroidism, and diabetes had a higher mean of abortion ratio. Also, in women with high blood pressure, heavy physical activity, and BMI < 18.9 as well as those who were living in rural areas, mean abortion ratio was less than the others (Figure 1).

## 4. Discussion

Although the total burden related to maternal abortion and miscarriage is less than 0.02% in Iran, the emotional complications of abortion (such as depression) as well as its physical complications may face the families and women with different psychosocial problems.

In the current research, the lifetime prevalence of spontaneous abortion was about 26% which is variable between 10 and 31% in different studies [24, 34, 35]. Despite the fact that the prevalence of abortion in this study was consistent with the previous researches, this result cannot be generalized to the whole society. In fact, abortion may occur in the early weeks of pregnancy when the mother is not aware of her pregnancy. In addition, in countries such as Iran in which the induced abortion is illegal, it is not easy to know about the exact burden of different types of abortion. In such situation, women hide the exact reason of abortion in order to take the advantage of hospital care.

The findings of this study suggested that age at the first marriage and age at the first pregnancy are important risk factors in spontaneous abortion. Accordingly, the risk for abortion in women with the first marriage and pregnancy at age greater than 26 years old was 57% and 87% more than the other age groups, respectively. This finding was also consistent with previous researches [5, 36]. Scientists believe that marriage and pregnancy of a mother at older ages increase the risk of abortion, fetal and chromosomal problems, and pregnancy-related complications [37]. Therefore, as it has been recommended, it is necessary to have regular check-ups and tests on the natural development of fetuses in pregnant women of older ages.

According to the findings of this research, secondhand smoking increased the risk for spontaneous abortion but not significantly. However, in similar studies, there was a significant relationship between increased abortion risk and secondhand smoking [23, 38]. There is no single stage at which smoking is safe; thus, pregnant women should keep themselves away from exposure to tobacco contamination.

Contraceptive pills, as a preventive factor, decreased the abortion risk by 78% which was consistent with the findings of similar studies [39–41]. It may be because the contraceptive pills also have therapeutic effects, in addition to the contraceptive effect, and they are sometimes used to prevent ovarian cysts or to strengthen the follicles [42, 43]. There is also another therapeutic way to reduce abortion risk: the use of progesterone hormones prescribed by a gynecologist. Therefore, some hormonal contraceptive methods that contain progesterone may play a role in preventing spontaneous abortion [44].

In line with the findings of previous studies, the risk for abortion increased with the increasing number of pregnancies [8, 45, 46].

The odds for spontaneous abortion increased with increasing level of education so the women with secondary education were at the greatest risk for spontaneous abortion. Many studies concluded that negative consequences of pregnancies were more evident in women with higher education [47, 48]; however, a research in the northwest Ethiopia reached a conflicting result [36]. As women with secondary education decide to get married at older ages, factors such as older age at the first marriage as well as first pregnancy—a risk factor for spontaneous abortion—can increase the risk for abortion.

As shown in this study, the odds of abortion increased with the increasing SES. After adjusting the variables, it was found that pregnant women with higher SES are 1.36 times more likely to have abortion. In many of the previous studies, significant relationship was found between spontaneous abortion and SES [26, 49].

The prevalence of spontaneous abortion in women with hypertension was greater than in those without hypertension, and only 6.2% of the subjects who experienced spontaneous abortion had high blood pressure. After adjustment for other variables, it was found that women with high blood pressure were at a lower risk for spontaneous abortion. In the study conducted in Finland, no significant correlation was found between blood pressure and spontaneous abortion [35], and such finding might be due to the fact that hypertensive mothers get better health care which in turn helps to prevent abortion.

One of the limitations of this study was employing the self-report questionnaires to be completed by the subjects which may cause a problem in identifying spontaneous and nonspontaneous abortions. In Iranian culture and in area where people are still stick to the traditions such as where Kurdish people are living, families and women feel ashamed for any type of abortion. In addition, the induced abortion is illegal if there is no medical justification approved by forensic medicine and specialist. Such regulation contributes to not having an exact view regarding the true prevalence and the types of abortion. However, in the RaNCD cohort study, the investigator in line with the protocol of the PERSIAN cohort used a local and female interviewer for women, in order to get the correct answers to the questions. Such interviewer reassured the participants regarding the confidentiality of provided answers.

## 5. Conclusion

For countries such as Iran in which the psychosocial complication of abortion might be prominent, it is of great importance to know its risk factors within a population-based study. According to our results, as the number of pregnancies, age at the first marriage, age at the first pregnancy, and education level increase, the risk for spontaneous abortion also increases. While in line with socioeconomical development of women in Iran, all of such factors are increasing over the recent years, preventing abortion among such women in Iran is of great importance. In fact, strategies should be implemented through mass media, counseling, further education, and training about abortion and its risk factors to both people and medical care providers. While primary health care and maternal care given by both midwife and gynecologists have been provided for most of the cities all around Iran, a more high-quality care is needed for high-risk women.

## Figures and Tables

**Table 1:** Descriptive statistics of the sampled population and the results of the logistic regression model for risk factors of spontaneous abortion.

Variables	Total	Abortion	Abortion ratio	Adjusted
	*N* (%)	*N* (prevalence)	Mean (SD)	OR (95% CI)
Total (%)	4831 (100)	1241 (25.7)	0.10 (0.2)	
Menstruation start age				
<12 years	1031 (21.4)	276 (26.8)	0.11 (0.2)	1
13-16 years	3472 (72.2)	891 (25.7)	0.11 (0.2)	0.9 (0.8-1.1)
>17 years	306 (6.4)	64 (20.9)	0.07 (0.1)	0.7 (0.5-1.0)
Pregnancy number				
1-3	1799 (37.3)	255 (14.2)	0.09 (0.2)	1
4-5	1376 (28.5)	420 (30.5)	0.14 (0.3)	4.5 (3.7-5.5)
≥6	1648 (34.2)	558 (33.8)	0.10 (0.2)	8.3 (6.6-10.5)
First pregnancy age (year)				
15	611 (12.7)	169 (27.6)	0.08 (0.2)	1
15-20	2469 (51.2)	620 (25.1)	0.09 (0.2)	0.9 (0.8-1.2)
21-25	1159 (24.1)	286 (24.7)	0.11 (0.2)	1.3 (0.9-1.6)
≥26	581 (12.1)	155 (26.7)	0.20 (0.4)	1.9 (1.3-2. 8)
First marriage age (year)				
≥15	1406 (29.1)	375 (26.7)	0.08 (0.2)	1
16-20	2338 (48.4)	593 (25.4)	0.10 (0.2)	1.2 (0.9-1.4)
21-25	728 (15.1)	174 (23.9)	0.12 (0.2)	1.2 (0.9-1.6)
≥26	358 (7.4)	98 (27.4)	0.19 (0.4)	1.6 (1.0-2.4)
Level of education				
Illiterate	1791 (37.3)	433 (24.1)	0.06 (0.1)	1
1-5 years	2202 (45.9)	582 (26.4)	0.12 (0.3)	1.8 (1.5-2.1)
6-9 years	461 (9.6)	123 (26.8)	0.14 (0.3)	2.4 (1.8-3.2)
10-12 years	243 (5.1)	62 (25.5)	0.18 (0.4)	2.4 (1.6-3.5)
≥13 years	111 (2.1)	41 (36.9)	0.23 (0.3)	3.5 (2.1-5.8)
Place				
Urban	2083 (58.0)	772 (34.7)	0.12 (0.3)	1
Rural	1507 (42.0)	469 (31.1)	0.09 (0.2)	0.9 (0.8-1.1)
Consanguineous marriage				
No	3608 (74.8)	934 (25.9)	0.10 (0.2)	1
First degree	678 (14.1)	172 (25.4)	0.11 (0.3)	0.9 (0. 8-1.2)
Second degree	541 (11.1)	135 (2.9)	0.09 (0.2)	0.9 (0. 8-1.2)
Smoking status				
No	4548 (94.4)	1166 (25.6)	0.11 (0.2)	1
Current	105 (2.2)	22 (20.1)	0.05 (0.1)	0.7 (0.4-1.2)
Former	165 (3.4)	48 (29.1)	0.07 (0.1)	1.2 (0.8-1.7)
Secondhand smoking				
No	2446 (50.6)	599 (24.5)	0.10 (0.2)	1
Yes	2385 (49.4)	642 (26.9)	0.11 (0.3)	1.1 (1.0-1.3)
BMI				
<18.9	52 (1.1)	7 (14)	0.09 (0.2)	1
19-24.9	965 (20.1)	229 (23.7)	0.11 (0.2)	1.9 (0.8-4.6)
25-29.9	1952 (40.6)	504 (25.8)	0.12 (0.2)	2.2 (0.96-4.9)
30-34.9	1381 (28.8)	380 (27.5)	0.09 (0.2)	2.4 (1.1-5.5)
>35	452 (9.4)	115 (25.4)	0.9 (0.2)	2.3 (0.9-5.3)
Physical activity daily METs				
24-36.5	1036 (21.4)	261 (25.2)	0.11 (0.3)	1
36.6-44.9	3302 (68.4)	862 (26.1)	0.11 (0.2)	1.1 (0.9-1.3)
≥45	492 (10.2)	118 (23.9)	0.08 (0.2)	1.0 (0. 8-1.4)
Use contraceptive drug				
No	812 (16.8)	240 (29.5)	0.13 (0.3)	1
Yes	4013 (83.2)	996 (24.8)	0.10 (0.2)	0.8 (0.6-0.9)
Socioeconomic status				
1st quantile (the poorest)	968 (20.1)	236 (24.4)	0.08 (0.2)	1
2nd quantile	966 (20.0)	222 (22.9)	0.09 (0.2)	0.9 (0.7-1.1)
3rd quantile	962 (19.9)	242 (25.1)	0.09 (0.2)	1.0 (0.8-1.3)
4th quantile	968 (20.1)	248 (25.6)	0.11 (0.3)	1.0 (0.8-1.3)
5th quantile (the richest)	962 (19.9)	291 (30.2)	0.15 (0.3)	1.4 (1.1-1.8)
Thyroid				
No	4546 (94.1)	1159 (25.5)	0.10 (0.3)	1
Hypo	261 (5.4)	73 (27.9)	0.1 (0.2)	1.1 (0.8-1.5)
Hyper	24 (0.5)	9 (37.5)	0.14 (0.2)	1.8 (0.7-4.5)
Blood pressure				
No	4465 (92.7)	1158 (25.9)	0.11 (0.2)	1
Yes	353 (7.33)	76 (21.5)	0.12 (0.3)	0.6 (0.5-0.8)
Goodness of fit model				
Sensitivity	19.15%			
Specificity	95.25%			
Accuracy	75.97%			
Positive predictive value	57.79%			
Negative predictive value	77.63%			

## Data Availability

All the information on how to access the RaNCD, with a list of current proposals and papers currently under preparation, can be found on our website: http://www.persiancohort.com.

## Abbreviations

PCA: Principal component analysis
SES: Socioeconomic status
RaNCD: Ravansar Non-Communicable Disease
HEI: Healthy Eating Index.
